# Spatiotemporal dynamics of bile acid profiles in broilers from hatch to market age

**DOI:** 10.1016/j.psj.2026.107353

**Published:** 2026-06-27

**Authors:** Sunlin Luo, Ruiqi Tan, Qiaomin Duan, Wenjun He, Ying Liu, Yiqiang Chen

**Affiliations:** State Key Laboratory of Animal Nutrition and Feeding, College of Animal Science and Technology, China Agricultural University, Beijing 100193, China

**Keywords:** Bile acid profile, Enterohepatic circulation, Developmental dynamics, Broiler

## Abstract

Systematic research on the dynamic distribution of bile acids (BAs) throughout the enterohepatic axis in broilers remains limited. Therefore, this study aimed to elucidate the spatiotemporal dynamics of the BA pool in broilers from hatching to market age. Newly hatched broiler chickens were reared under standard conditions for 42 days. At the end of each week, six birds were randomly selected to collect samples for the determination of BAs. The results revealed a clear spatial concentration gradient of total bile acids (TBAs). Bile exhibited the highest TBA concentration (approximately 10⁸ ng/mL), followed by small intestine and colorectum (approximately 10⁶–10⁷ ng/g DM), liver (approximately 10⁵–10⁶ ng/g DM), cecum (< 10⁵ ng/g DM), and serum (approximately 10³ ng/mL). The ileum and cecum were identified as active sites for microbial BA metabolism. Microbial deconjugation was primarily observed in the ileum. There was a significant increase in the proportion of unconjugated BAs, rising from 48.35% to 83.92% by week 3 (*P* < 0.01). Concurrently, microbial 7α-dehydroxylation was prevalent in the cecum. The proportion of secondary BAs, primarily lithocholic acid (LCA), increased progressively, with a significant rise observed by week 3 (*P* < 0.05). These findings indicate that week 3 may be a critical period for the functional maturation of BA-metabolizing microbiota. Principal component analysis (PCA) and permutational multivariate analysis of variance (PERMANOVA) analyses confirmed significant spatial heterogeneity in BA profiles across the enterohepatic axis (*P* = 0.001). The liver, bile and serum maintained stable profiles dominated by taurine-conjugated primary BAs. Limited microbial deconjugation occurred in the duodenum and jejunum. The cecum consistently formed a distinct cluster characterised by a significantly higher proportion of secondary BAs. Notably, no significant difference was found between the BA profiles of the ileum and colorectum (*P* > 0.05). This finding highlights a species‑specific aspect of avian digestive physiology, differing from the clear separation between ileal and colon bile acid profiles typically seen in mammals.

## Introduction

Bile acids (BAs) are important amphipathic molecules that are derived from the metabolism of hepatic cholesterol (Chiang, 2013). They act as efficient biological detergents, emulsifying dietary fats and fat-soluble vitamins within the intestine to form mixed micelles that facilitate their absorption (Poland and Flynn, 2021). This function is crucial for rapidly growing broiler chickens, as efficient lipid utilisation is fundamental to achieving optimal growth performance and feed conversion ratio. Indeed, it has been suggested that low fat utilisation efficiency in chicks may be associated with relative BA insufficiency in the early stages of life (Renner and Hill, 1960; Kussaibati et al., 1982). In China, porcine-derived BAs are approved as feed additives for broilers, improving growth performance and enhancing fat utilisation (Geng et al., 2022; Hu et al., 2024; Liu et al., 2025). This highlights the direct impact of BA composition and function on livestock production.

BAs can be classified into several categories based on their metabolic origin and chemical structure. Primary BAs are synthesized in the liver from cholesterol, whereas secondary BAs are produced by gut microbial metabolism of primary BAs in the intestine (Kiriyama and Nochi, 2021). Conjugated bile acids refer to those in which the side-chain carboxyl group is amidated with amino acid (mainly glycine or taurine), while unconjugated BAs lack such conjugation (Hofmann and Mysels, 1992). In addition, BAs can be categorized by the presence or absence of a hydroxyl group at the C‑12 position: 12α‑hydroxylated (12α-OH) BAs (e.g., cholic acid, CA) have a hydroxyl group at C‑12, whereas non‑12α‑hydroxylated (non-12α-OH) BAs (e.g., chenodeoxycholic acid, CDCA) do not (Fan et al., 2025). The physiological effects of BAs are intricately linked to the composition of specific categories within the pool (Fan et al., 2025). For example, a high proportion of secondary BAs has been associated with increased body fat in obese pigs (Hu et al., 2024). Similarly, increasing the proportion of conjugated BAs in the small intestine of broilers through dietary xylanase and β‑glucanase has been shown to enhance fat absorption (Mathlouthi et al., 2002). Therefore, a detailed understanding of the composition of specific BA categories is pivotal for devising interventions to improve animal production.

In recent years, research has revealed that the functions of BAs extend far beyond physical digestion. These important signalling molecules can broadly regulate poultry lipid metabolism and muscle growth by activating nuclear receptors farnesoid X receptor (FXR) and membrane receptors Takeda G protein-coupled receptor 5 (TGR5) (Chen et al., 2024; Jia and Dong, 2024; Zhang et al., 2025). At the same time, the composition of BAs has a significant impact on the composition of the gut microbiota. The gut microbiota transforms BAs through a variety of enzymes, including bile salt hydrolase and 7α-dehydroxylase, via processes such as deconjugation, dehydroxylation, oxidation and isomerisation (Ridlon et al., 2016). These transformations not only alter the physicochemical properties of BAs (deconjugation and dehydroxylation significantly increase their hydrophobicity and potential toxicity) but also generate secondary BAs with distinct biological activities, thereby constituting a complex host-microbiota metabolic dialogue (Monte et al., 2009; Collins et al., 2023). Consequently, the size and composition of the BA pool are central to maintaining metabolic health and intestinal homeostasis.

The current systematic understanding of BAs is primarily derived from experimental animal models (Yang et al., 2017; Duan et al., 2022). Relatively little research has been conducted on livestock animals, particularly broilers (Jia and Dong, 2024). Early work using an enzymatic method measured total BA concentrations in the serum, bile, jejunal contents and excreta of broilers during the first six weeks (Green and Kellogg, 1987). However, such enzymatic assays can only quantify total BAs and cannot distinguish individual BA species (Yang et al., 2017). Consequently, the early study was unable to delineate complex BA profiles or reveal their dynamic changes and spatial distribution. Although liquid chromatography‑tandem mass spectrometry (LC‑MS/MS) now enables the identification and quantification of individual BA species, recent studies on broilers remain limited in scope. One study compared the composition of BAs across six species, including chickens, and revealed significant differences between species (Qi et al., 2024). Another study investigated the impact of various diets on the concentrations of total BAs and four high-abundance BAs in the circulation and intestine of broilers (Buyse et al., 2026). Thus, a systematic spatiotemporal study of BA profiles from hatch to market age (weekly changes from d 7 to d 42) across multiple compartments (liver, bile, serum, and different intestinal section contents) in broilers is still lacking.

Therefore, this study aims to investigate the dynamic changes in broiler BA profiles from hatch to market age (weekly changes from d 7 to d 42) through longitudinal multi-point sampling. This study will provide essential foundational data to improve our understanding of the development of digestive physiology and production performance in broilers through nutritional strategies.

## Materials and methods

### Animal ethics statement

All animal experimental procedures applied in this study were reviewed and approved by the Animal Welfare Committee of China Agricultural University (approval number AW40704202-1-6, Beijing, China). This study was performed in the Fengning Research Unit of China Agricultural University (Chengde, Hebei, China).

### Animal management

A total of 72 one-d-old Arbor Acres (AA) male broilers (42.93 ± 0.57 g) were obtained from the Hebei Kangyu Poultry Breeding Co., Ltd. (Langfang, China) and were divided into six replicates with 12 broilers per replicate. All broilers were fed a basal diet. The diets were formulated based on the nutritional requirements for broilers recommended by the Chicken Feeding Standard of China (NY/T 33-2004). The composition and nutritional levels of the basal diets as shown in Table 1. Crude protein was determined by the Kjeldahl method. Calcium was determined by potassium permanganate titration. Total phosphorus was determined by spectrophotometry. Lysine was measured using an amino acid analyzer after acid hydrolysis. Methionine and cysteine were measured using an amino acid analyzer after oxidative hydrolysis. Animals were housed in suitable, controlled environments, and they had free access to standard feed and clean water. During the initial week, the temperature was maintained at 34°C, subsequently decreasing at a rate of 2°C per week until it reached 24°C. The relative humidity was maintained at 45% to 55%. The experiment was conducted over a 42-day period, with a 24-hour light programme being applied during the initial 3 days. This was followed by a 23-hour light to 1-hour dark lighting programme, which was utilised until d 42. Immunization and feeding management were performed according to the standard immunization procedures for broilers.

**Table 1:** Experimental diet composition (as-fed basis, %).

Ingredients	Starter phase (d 0 to 21)	Grower phase (d 22 to 42)
**Ingredients**		
Corn	60.13	61.53
Soybean meal	32.50	31.70
Fish meal	2.00	0.00
Soybean oil	1.50	3.00
Dicalcium phosphate	1.50	1.70
Limestone	1.34	1.15
DL-methionine (98 %)	0.23	0.12
Salt	0.30	0.30
Premix^1^	0.50	0.50
Total	100.00	100.00
**Nutrients**^2^		
Metabolizable energy, MJ/kg	12.59	13.22
Crude protein	21.16	19.62
Calcium	1.02	0.96
Total phosphorus	0.63	0.60
Lysine	1.17	1.08
Methionine + Cysteine	0.92	0.76

1 The premix provided the following vitamins and minerals per kilogram of feed: Vitamin A, 9,000 IU; Vitamin D_3_, 3,000 IU; Vitamin E, 24 mg; Vitamin K_3_, 1.8 mg; Vitamin B_1_, 2.0 mg; Riboflavin, 5.0 mg; Vitamin B_6_, 3.0 mg; Vitamin B_12_, 0.1 mg; Nicotinic acid, 40 mg; Pantothenic acid, 15 mg; Folic acid, 1.0 mg; Biotin, 0.05 mg; Choline chloride, 500 mg; Iron, 80 mg; Copper, 20 mg; Zinc, 90 mg; Iodine, 0.35mg; Selenium, 0.30 mg.

2 All of the nutritional data were analyzed except for metabolizable energy.

### Sample collection

On d 7, d 14, d 21, d 28, d 35, and d 42 of the experiment, six broilers (one per replicate, *n* = 6) were randomly selected and slaughtered for sample collection. All samplings were performed at 1 h post-feeding in the morning to minimize feeding status effects and ensure comparability of bile acid profiles across weeks. The selected broilers were not replaced, and no broiler was sampled more than once throughout the experiment. Blood was collected from the wing vein and centrifuged at 3,000 × *g* for 10 minutes to obtain serum. After blood collection, the broiler chickens were euthanized by severing their jugular veins. Bile, liver and intestinal contents samples were then collected immediately. For intestinal contents, full‑lumen digesta (without intestinal mucosa) were collected from the duodenum, jejunum, ileum, cecum, and colorectum of each broiler. The whole liver and all digesta from each intestinal segment were collected to ensure representative sampling. Liver and intestinal content samples were then freeze‑dried and homogenized before further analysis. All samples were stored at −20°C for further analysis.

### Quantitative analysis of bile acids

The concentrations of BAs in the samples were analysed using a validated LC-MS/MS method. For sample pretreatment, 50 µL of serum was mixed with 10 µL of an isotope internal standard solution (1.0 µg/mL) and extracted twice with 250 µL of methanol. Liver and intestinal content samples (20 mg each) were extracted three times with 500 µL of methanol. For bile samples (50 µL), extraction was performed three times using 1 mL of methanol. All samples were homogenised using a fully automatic sample freezing grinder (Shanghai Jingxin Industrial Development Co., Ltd., China) during the extraction process. The combined serum extracts were dried under nitrogen and redissolved in 100 µL of a 50% methanol and water solution. The extracts from the liver, intestinal contents and bile were diluted with 50% methanol in water. Then, 90 µL of the diluted solution was mixed with 10 µL of the isotope internal standard solution (1.0 µg/mL). All final solutions were then analysed using LC-MS/MS. All solid samples (liver and intestinal contents) were freeze-dried before extraction.

All samples were analysed by LC-MS/MS (Agilent technologies 1200 series high-performance liquid chromatography and Agilent 6460 triple quadrupole mass spectrometer, Agilent technologies, USA) after pretreatment. The samples were separated on a Waters Acquity UPLC BEH C18 ultra-high performance liquid chromatography column (1.7 μm, 2.1 × 100 mm). Individual bile acids were identified and quantified by comparing their retention times and peak area responses with those of authentic BA standards. The matrix effect was corrected using isotope internal standards. Gradient elution was performed using 0.1 % formic acid in water and 0.1 % formic acid in acetonitrile as mobile phases at a flow rate of 0.5 mL/min. Mass spectrometry was conducted in negative ion mode and multiple reaction monitoring. The mass spectrometry detection parameters and classification of BAs are presented in Table S1. The relative concentrations of different types of BAs represent the ratio of the concentration of each type of BA to the TBA concentration.

### Data analysis

Agilent MassHunter Workstation qualitative and quantitative analysis 10.0 software was used for data acquisition and processing. Univariate statistical analysis was performed using GraphPad Prism (version 10.6). Differences in total bile acid concentration and the relative proportions of BA classes were assessed by one-way analysis of variance (ANOVA). Two distinct post hoc comparison strategies were applied depending on the experimental design. For temporal comparisons (across weeks within the same tissue), one‑way ANOVA was performed, followed by Bonferroni‑adjusted pairwise comparisons between consecutive weeks. The resulting *P*‑values were adjusted to control the family‑wise error rate across the five sequential comparisons within each compartment. For spatial comparisons (between different tissues at the same age), one‑way ANOVA was performed, followed by Tukey’s honestly significant difference (HSD) test for all possible pairwise comparisons. Differences were considered statistically significant at adjusted *P* < 0.05 (Bonferroni for temporal, Tukey for spatial comparisons). All related graphical visualizations were generated using GraphPad Prism.

Multivariate statistical analysis of BA composition was performed using the MetaboAnalyst platform (version 6.0, https://dev.metaboanalyst.ca). Principal component analysis (PCA) was performed on the relative proportions (%) of individual BA species (normalized within each sample) to reduce dimensionality and visualize overall variation in BA profiles among different ages and sample types. To test the statistical significance of the observed separations, permutational multivariate analysis of variance (PERMANOVA) was conducted based on a Bray-Curtis dissimilarity matrix using 999 permutations. When a significant global effect was found (*P* < 0.05), pairwise comparisons between groups (e.g., between weeks for the same tissue, or between tissues at the same age) were performed using the same PERMANOVA model with 999 permutations. The resulting *P*-values from all pairwise tests were adjusted for multiple comparisons using the false discovery rate (FDR) method. An adjusted *P*-value (*P*-adj) of less than 0.05 was considered statistically significant, indicating a distinct BA composition between the two compared groups.

## Results

### Spatial distribution and developmental dynamics of total BA concentrations

**Fig. 1:**
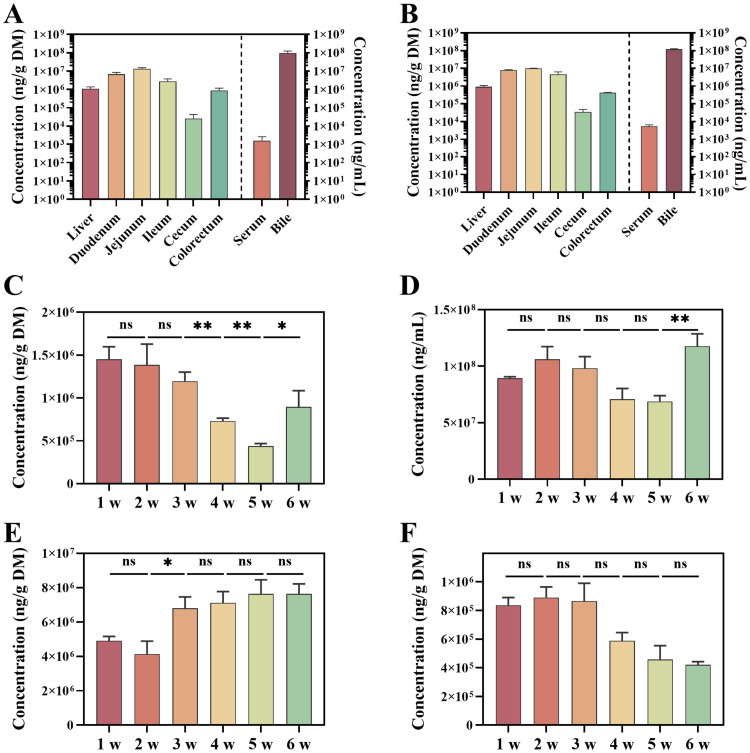
**Dynamic changes of total bile acid (TBA) concentrations in broiler tissues and biofluids across weeks of age**. Concentrations of TBA in liver, duodenum, jejunum, ileum, cecum, and colorectum, serum, and bile at (A) d 21 and (B) d 42. In panels (A) and (B), the vertical dashed line separates solid tissue samples (liver and intestinal contents; left side, unit: ng/g DM) from biofluid samples (serum and bile; right side, unit: ng/mL). Age-dependent trajectories of TBA concentrations in four key compartments, (C) liver, (D) bile, (E) duodenum, and (F) colorectum. Data are presented as mean ± SEM (*n* = 6). Analyze the differences between adjacent weeks through multiple comparisons. * *P* < 0.05, ** *P* < 0.01.

As shown in Fig. 1A and 1B for the two time points (21 and 42 days of age), gallbladder bile consistently exhibited the highest total BA (TBA) concentration, ranging from approximately 9 × 10⁷ to 1.2 × 10⁸ ng/mL. The small intestinal (duodenum, jejunum, and ileum) followed, with concentrations ranging from 2 × 10^6^ to 1.4 × 10^7^ ng/g DM. Liver tissue showed an intermediate concentration ranging from approximately 8 × 10^5^ to 1 × 10^6^ ng/g DM. Colorectum TBA concentration ranged from 4 × 10^5^ to 9 × 10^5^ ng/g DM. The cecum had the lowest TBA content within the gastrointestinal tract (approximately 2.5 × 10^4^ to 3.5 × 10^4^ ng/g DM), while serum levels were minimal (approximately 1 × 10^3^ to 5 × 10^3^ ng/mL).

The developmental trajectories of TBA concentration in the four pivotal compartments selected as key functional nodes of the enterohepatic circulation (the liver for synthesis, the bile for storage, the duodenum for secretion and digestion, and the colorectum for excretion) revealed distinct organ-specific patterns (Fig. 1C–F). Hepatic TBA concentration remained stable during the first three weeks, then declined significantly in weeks 4 and 5 (*P* < 0.01), before rebounding at week 6 (*P* < 0.05). In contrast, the concentration in bile showed no variation from weeks 1 to 5, before increasing sharply at week 6 (*P* < 0.01). Duodenal content exhibited relative stability, with a single elevation at week 3 (*P* < 0.05). Colorectum TBA output remained largely constant throughout the study (*P* > 0.05).

### Characterization and age-dependent dynamics of BA profiles in eight broiler compartments

**Fig. 2:**
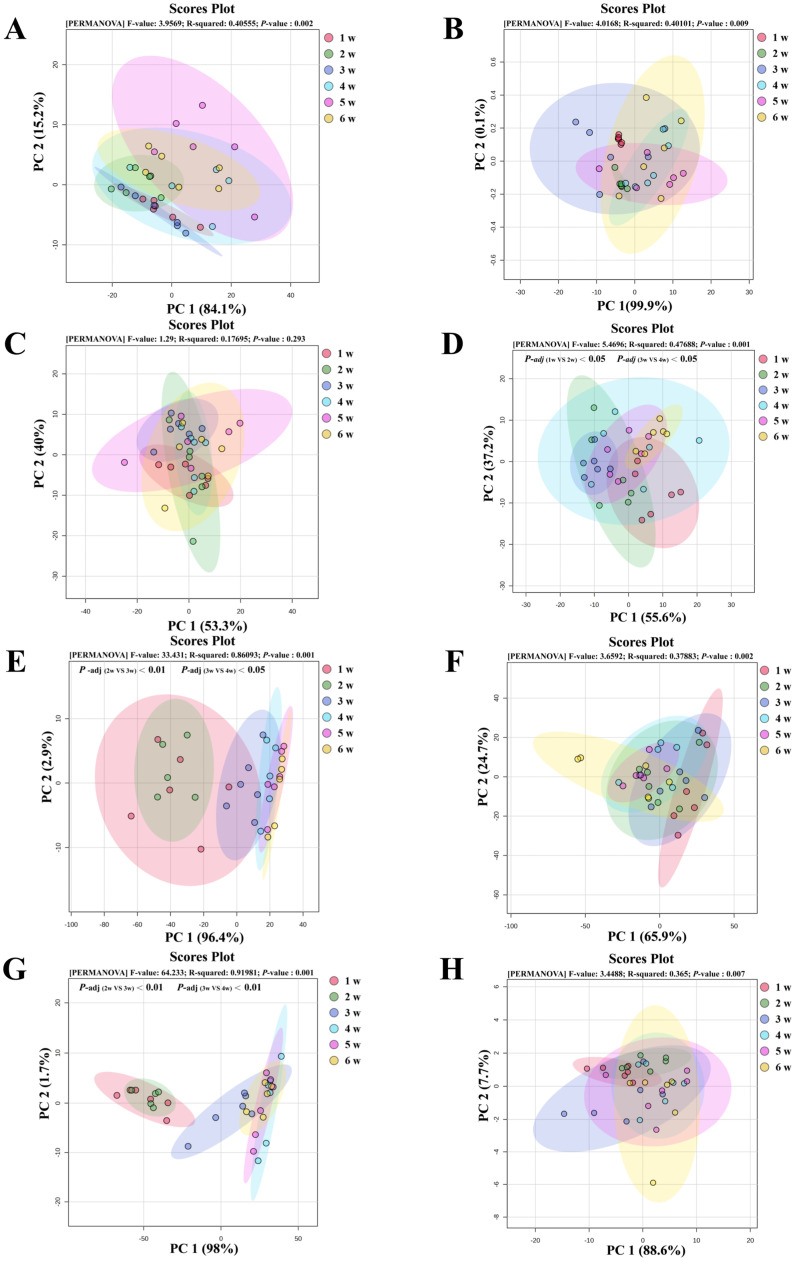
**Principal component analysis (PCA) reveals weekly transitions in the bile acid profiles of broilers**. PCA plots with 95% confidence ellipses illustrate the weekly compositional changes. The significant divergence between adjacent weeks (*P* < 0.05, PERMANOVA pairwise comparison). (A) Liver, (B) Bile, (C) Duodenum, (D) Jejunum, (E) Ileum, (F) Cecum, (G) Colorectum, and (H) Serum. *n* = 6.

**Fig. 3:**
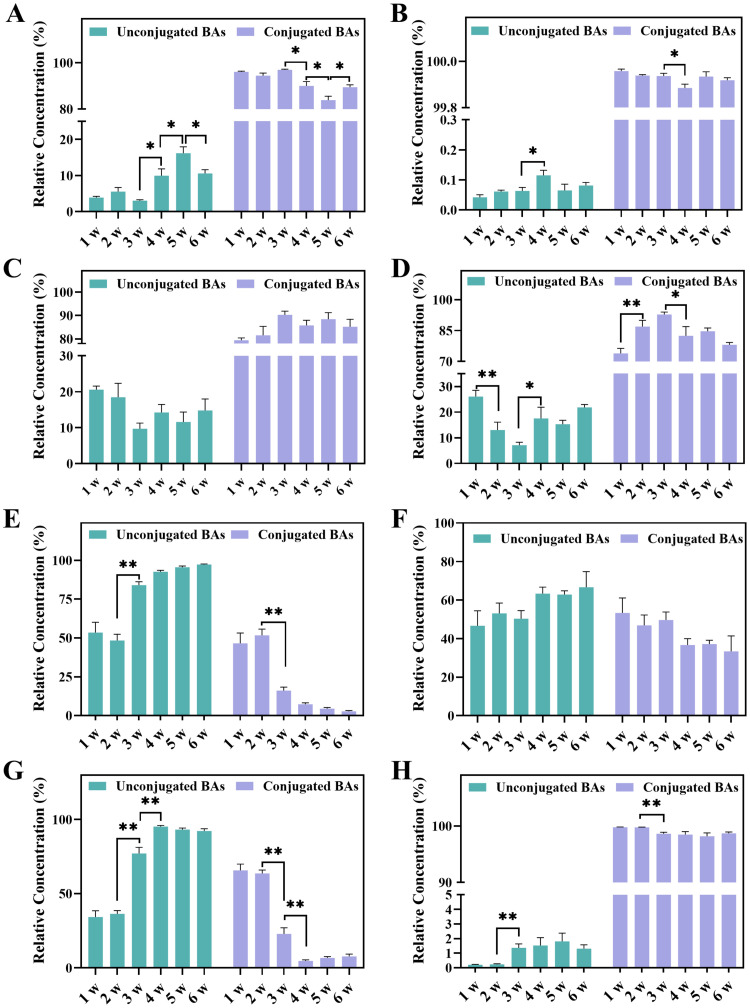
**Dynamic changes of conjugated and unconjugated bile acids (BAs) in broiler tissues and biofluids across weeks of age**. (A) Liver, (B) Bile, (C) Duodenum, (D) Jejunum, (E) Ileum, (F) Cecum, (G) Colorectum, and (H) Serum. For each tissue, one‑way ANOVA with Bonferroni‑adjusted pairwise comparisons was performed between consecutive weeks. Data are presented as mean ± SEM (*n* = 6). * *P* < 0.05, ** *P* < 0.01.

**Fig. 4:**
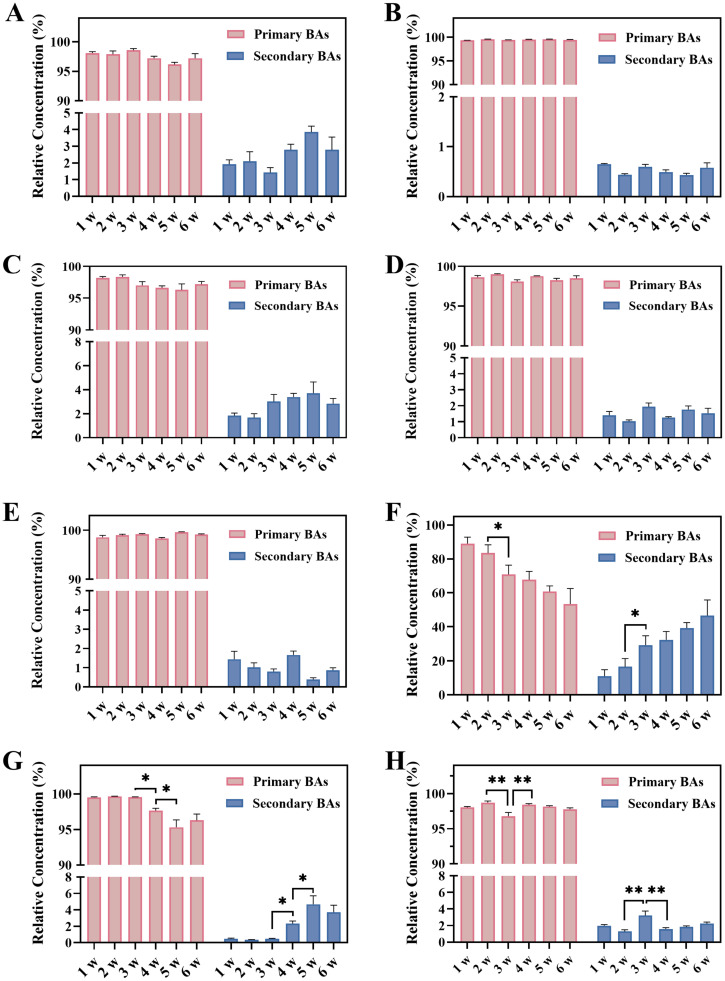
**Dynamic changes of primary and secondary bile acids (BAs) in broiler tissues and biofluids across weeks of age**. (A) Liver, (B) Bile, (C) Duodenum, (D) Jejunum, (E) Ileum, (F) Cecum, (G) Colorectum, and (H) Serum. For each tissue, one‑way ANOVA with Bonferroni‑adjusted pairwise comparisons was performed between consecutive weeks. Data are presented as mean ± SEM (*n* = 6). * *P* < 0.05, ** *P* < 0.01.

**Fig. 5:**
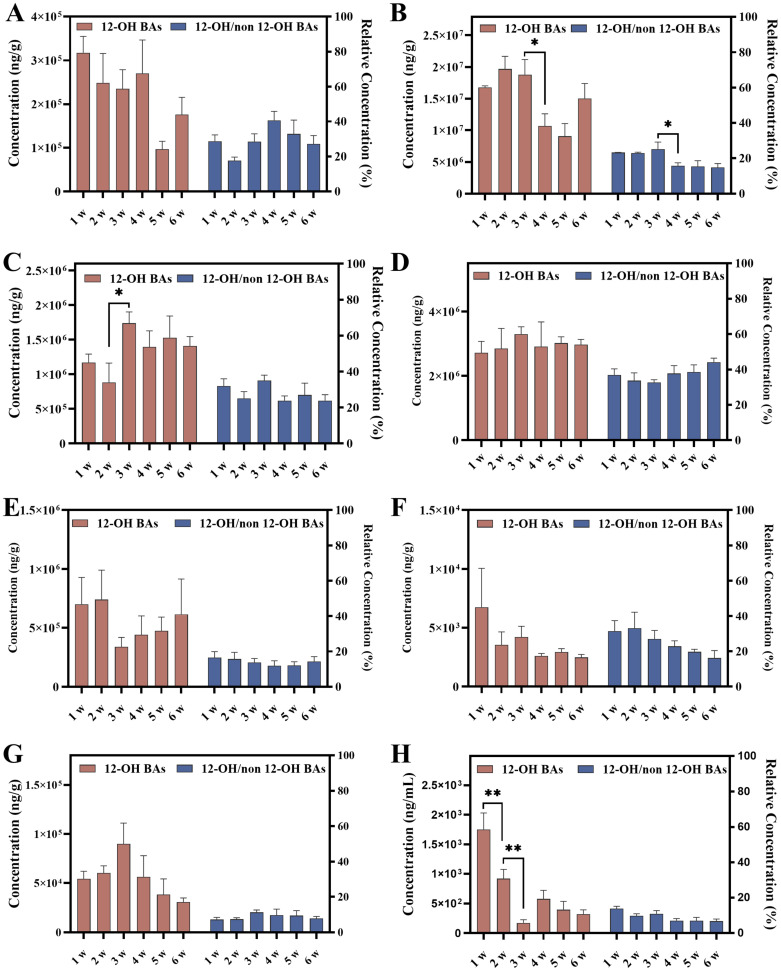
**Dynamic changes of the concentrations of 12α-hydroxylated (12-OH BAs) and the relative proportions of 12-OH BAs and non-12α-hydroxylated bile acids (non-12OH BAs) in broiler across weeks of age**. (A) Liver, (B) Bile, (C) Duodenum, (D) Jejunum, (E) Ileum, (F) Cecum, (G) Colorectum, and (H) Serum. For each tissue, one‑way ANOVA with Bonferroni‑adjusted pairwise comparisons was performed between consecutive weeks. Data are presented as mean ± SEM (*n* = 6). * *P* < 0.05, ** *P* < 0.01.

PERMANOVA revealed a progressive and significant change in the BA profile of broiler liver, with no significant differences in the BA profile of broiler livers between adjacent weeks (Fig. 2A; F = 3.96, *P* = 0.002). Taurochenodeoxycholic acid (TCDCA) and taurocholic acid (TCA) were the predominant species throughout the study, accounting for over 85% of the total hepatic BAs. Notably, the proportion of TCDCA remained above 60% throughout the study (Fig. S1A). Conjugated BAs were the dominant form in the liver. However, a significant increase in the proportion of unconjugated BAs, accompanied by a decrease in the proportion of conjugated forms, was observed specifically at weeks 4 and 5 (Fig. 3A, *P* < 0.05). Meanwhile, the proportion of chenodeoxycholic acid (CDCA) in the liver increased in the fourth and fifth weeks (Fig. S2A, *P* < 0.01). Primary BAs dominated the hepatic profile by over 95% throughout (Fig. 4A), while secondary BAs such as lithocholic acid (LCA) were present only in small amounts (Fig. S1A). The ratio of 12-OH BAs to non-12-OH BAs is related to glycolipid metabolism and nutrient absorption. The ratio of 12-OH BAs to non-12-OH BAs in broiler livers remained stable throughout the rearing period (approximately 20%-40%) (Fig. 5A).

PERMANOVA analysis revealed a progressive and significant change in the BA profile of broiler bile, with no significant differences observed in any pairwise comparisons between consecutive weeks (Fig. 2B; F=4.02, *P*=0.009). TCDCA and TCA were dominant, accounting for over 99% of the biliary pool, with TCDCA consistently exceeding 75% (Fig. S1B). As expected for a storage compartment of newly synthesised and recirculated BAs, unconjugated and secondary BAs were virtually absent. Conjugated BAs consistently accounted for over 99.8% of the total. A significant increase in the proportion of unconjugated BAs and a decrease in the proportion of conjugated forms were observed in week 4 compared to week 3 (Fig. 3B, *P* < 0.05). Primary BAs predominated at a proportion greater than 99%, and there was no age-related trend in their proportion (Fig. 4B, *P* > 0.05). Notably, the concentration of 12-OH BAs decreased significantly in week 4, as did the ratio of 12-OH BAs to non-12-OH BAs (Fig. 5B, *P* < 0.05). Meanwhile, there was a significant increase in the proportion of TCDCA (non-12-OH BA) and a decrease in the proportion of TCA (12-OH BA) in the bile by week four (Fig. S2B and C, *P* < 0.05).

PERMANOVA revealed no significant age-related alterations in the duodenal BA profile over the six-week period (Fig. 2C; F=1.29, *P*=0.29). TCDCA remained the predominant BA, maintaining a stable proportion of approximately 65% throughout the period, followed by TCA at around 20% (Fig. S1C). The major unconjugated BA was CDCA, which accounted for 6% to 13% of the total (Fig. S1C). As in the liver and bile, the duodenal BA pool was dominated by conjugated and primary BAs. Conjugated BAs accounted for 80%–90% of the total (Fig. 3C), while primary BAs consistently exceeded 96% (Fig. 4C). Although the concentration of 12-OH BAs increased significantly in week 3 compared to week 2 (*P* < 0.05), the ratio of 12-OH BAs to non-12-OH BAs remained stable, showing no significant variation from week to week (Fig. 5C).

In jejunum, the BA profile showed significant temporal dynamics (Fig. 2D; F=5.47, *P*=0.001), with distinct compositional alterations observed between weeks 1 and 2 and between weeks 3 and 4 (Fig. 2D, *P*-adj < 0.05). The proportion of unconjugated BAs decreased significantly in week 2 compared with week 1 (Fig. 3D, *P* < 0.01) and increased significantly in week 4 compared with week 3 (Fig. 3D, *P* < 0.05). These alterations can be attributed to changes in the efficiency of microbial deconjugation of taurine-conjugated BAs (TCDCA and TCA) in the jejunum. Specifically, it was observed that the proportion of TCA converted to CA decreased in week 2 compared to the week 1 (Fig. S2E and S2G), while the proportion of TCDCA converted to CDCA increased in week 4 compared to the week 3 (Fig. S2D and S2F). Conjugated BAs remained dominant in jejunum, consistently constituting over 70% of the BAs pool. The two most abundant BAs are TCDCA and TCA in jejunum (Fig. S1D). Primary BAs also predominated (> 97%), with no age-related trend (Fig. 4D, *P* > 0.05). The ratio of 12-OH BAs to non-12-OH BAs ranged from 32% to 44% during the observation period and remained stable over time (Fig. 5D).

The ileal BA profile showed clear age-related changes (Fig. 2E; F = 33.43, *P* = 0.001). During the first two weeks, the proportions of TCDCA and its unconjugated form, CDCA, were comparable (both approximately 41%-45%, Fig. S1E). A significant compositional transition occurred in week 3, acting as a critical turning point. Therefore, week 3 may represent a critical period for the maturation of microbial bile salt hydrolase (BSH) activity in the ileum. At this stage, the proportion of CDCA surged to 72% (Fig. S2H), while the proportion of TCDCA decreased markedly to approximately 14% (Fig. S2J). From week 4 onward, the proportion of CDCA stabilized at over 80% of the ileal BA pool (Fig. S2H), whereas the proportion of TCDCA continued to decline, reaching below 10% (Fig. S2J). A significant alteration in the BA profile was observed between weeks 2 and 3 (Fig. 2E, *P-adj* < 0.01). As expected, the proportion of unconjugated BAs was significantly higher in week 3 than in week 2 (Fig. 3E, *P* < 0.05). Notably, CA and TCA did not contribute to this alteration, as their proportions remained stable throughout the observation period (Fig. S2I and K). The change was driven by increased microbial deconjugation efficiency for TCDCA in week 3, as evidenced by a sharp decrease in the proportion of TCDCA (Fig. S2J) and a concurrent increase in the proportion of CDCA (Fig. S2H). A further significant change in the BA profile was observed between weeks 3 and 4 (Fig. 2E, *P-adj* < 0.05). After week 4, the proportion of unconjugated BAs continued to increase, reaching 97.22% by week 6, although no significant differences were detected between subsequent adjacent weeks (Fig. 3E). The ileal BA pool was consistently dominated by primary BAs, accounting for over 98% of the total throughout the study (Fig. 4E). The relative ratio of 12-OH BAs to non-12-OH BAs in the ileum showed no significant temporal changes during the observation period (Fig. 5E).

The cecal BA profile in broilers showed significant and progressive changes from week 1 to week 6. PERMANOVA revealed a significant effect of age on BA composition (Fig. 2F; F = 3.66, *P* = 0.002). However, this temporal change was gradual, as evidenced by the lack of statistically significant differences in all pairwise comparisons between adjacent weeks. The three components that consistently dominated the pool were TCDCA, CDCA and LCA (Fig. S1F). These three components accounted for over 80% of the total, and their relative prominence changed over time. In week 1, TCDCA was the most abundant component, with CDCA in second place. LCA represented less than 6% of the total. From weeks 2 to 4, CDCA became the predominant and stable component, while TCDCA continued to decline and LCA increased progressively. By weeks 5 and 6, the proportion of CDCA had decreased and that of LCA had increased further, with LCA becoming the most abundant BA in the cecum (Fig. S1F; Fig. S2L–N). A distinctive feature was the consistent presence of glycine-conjugated BAs, including glycochenodeoxycholic acid (GCDCA) and glycodeoxycholic acid (GDCA), which constituted 5% to 9% of the cecal BA pool. The proportions of unconjugated and conjugated BAs were comparable and showed no significant age-related trend (Fig. 3F). As expected, the proportion of secondary BAs increased continuously, primarily due to the rise in LCA, with a significant increase observed between weeks 2 and 3 (Fig. 4F, *P* < 0.05). This transition is indicative of a possible functional onset of microbial 7α‑dehydroxylase activity, and week 3 may likewise represent a key maturation period for this enzyme. The ratio of 12-OH BAs to non-12-OH BAs in the cecum remained stable throughout the observation period with no significant temporal change (Fig. 5F).

PERMANOVA revealed significant temporal changes in colorectum BA composition over the six-week period (Fig. 2G; F = 64.23, *P* = 0.001). Specifically, an abrupt transition was observed between weeks 2 and 3, and between weeks 3 and 4 (Fig. 2G; *P*-adj < 0.01). The profile was predominantly composed of TCDCA and CDCA, together accounting for over 90% of the total. At weeks 1 and 2, TCDCA was the dominant BA (at around 60%), with CDCA accounting for around 30%. A critical transition occurred at week 3, characterized by a sharp decline in TCDCA and a marked increase in CDCA. From weeks 4 to 6, CDCA sustained a high proportion (> 80%) as the dominant BA, while TCDCA remained at a low level. Although CA and TCA collectively constituted less than 10%, their temporal trends mirrored those of CDCA and TCDCA, respectively (Fig. S1G; Fig. S2O–R). As expected, the proportion of unconjugated BAs in colorectum increased significantly in weeks 3 and 4 (Fig. 3G; *P* < 0.01). The colorectum BA pool was primarily conjugated during weeks 1 and 2, but turned predominantly unconjugated after week 3 (Fig. 3G). Secondary BAs were present only in trace amounts. From weeks 1 to 3, the proportion of secondary BAs in the pool did not exceed 0.4%. However, significant increases in the proportion of secondary BAs were observed in weeks 4 (2.34%) and 5 (4.68%), due to rising proportions of LCA, deoxycholic acid (DCA), and ursodeoxycholic acid (UDCA) (Fig. 4G; Fig. S2S–U). Throughout the observation period, the ratio of 12-OH BAs to non-12-OH BAs remained stable at between 7% and 11% (Fig. 5G).

PERMANOVA revealed significant temporal changes in the serum profile over six weeks (Fig. 2H; F = 3.45, *P* = 0.007). However, this change was gradual, with no significant differences detected in any pairwise comparisons between consecutive weeks. Strikingly, although the overall profile changed significantly, the relative proportion of no individual BA exhibited a statistically significant time-related trend. Instead, the compositional change appears to be the result of the cumulative effect of minor variations in the proportions of multiple BAs. TCDCA was the dominant BA in serum across all weeks, accounting for approximately 85% to 90%, followed by TCA (5% to 10%). All other BAs together constituted less than 5% (Fig. S1H). Conjugated BAs consistently represented over 98% of the serum BA pool. There was a notable increase in the proportion of unconjugated BAs from week 2 (0.24%) to week 3 (1.37%; *P* < 0.01), which then stabilised (Fig. 3H). Primary BAs predominated, consistently exceeding 96% of the total serum pool (Fig. 4H).

### The spatial heterogeneity of BAs in broilers

**Fig. 6:**
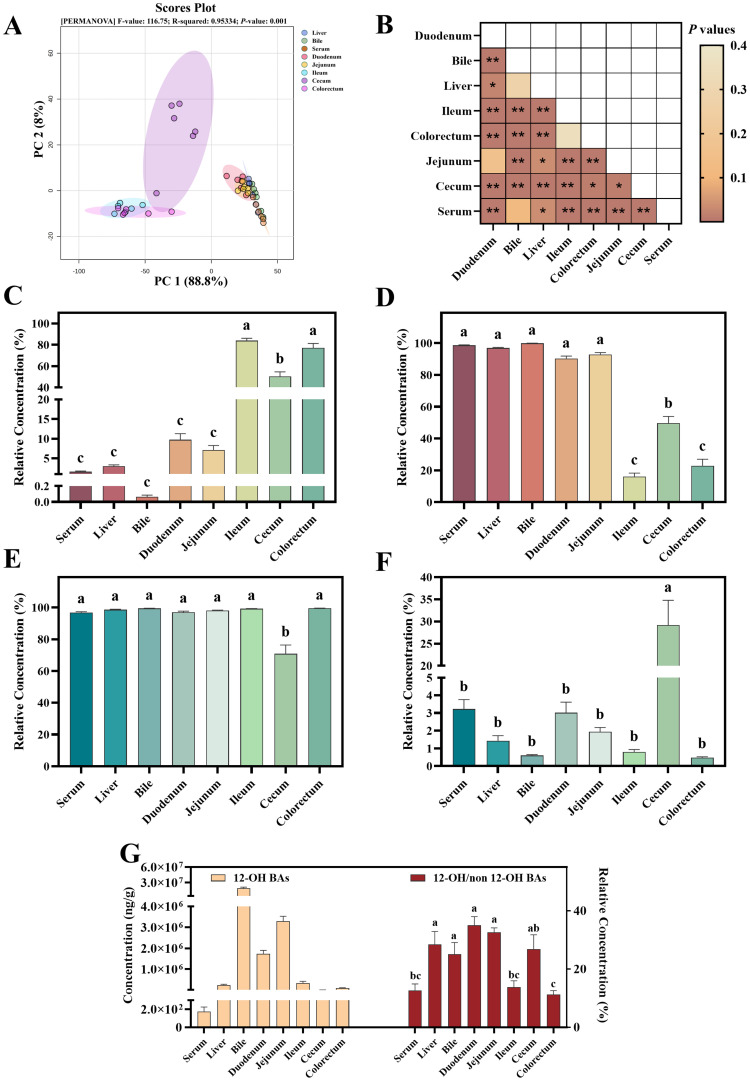
**The spatial heterogeneity of bile acids across the liver-gut axis samples in 21-day-old broilers**. (A) Principal component analysis (PCA) plot with 95% confidence ellipses depicting the overall compositional separation of bile acid profiles. (B) Heatmap of pairwise PERMANOVA *P*-values comparing bile acid composition between all sample types, * *P* < 0.05, ** *P* < 0.01. (C-F) Bar graphs showing the relative concentrations of key bile acid classifications in the samples: (C) unconjugated, (D) conjugated, (E) primary, and (F) secondary bile acids. (G) The concentrations of 12α-hydroxylated (12-OH BAs) and the relative proportions of 12-OH BAs and non-12α-hydroxylated bile acids (non-12OH BAs). For panels (C) through (G), different lowercase letters (a, b, c) above the bars indicate significant differences among sample types (*P* < 0.05, one‑way ANOVA followed by Tukey’s HSD test). Values sharing the same letter are not significantly different. Data are presented as mean ± SEM (*n* = 6).

To delineate the spatial distribution of BAs in broilers, we selected d 21 and d 42 as two key comparative time points. PCA was performed based on the relative proportions of individual bile acid species in each compartment. At d 21, significant spatial heterogeneity was evident across the eight compartments (Fig. 6A–B; F = 116.75, *P* = 0.001). The PCA plot (Fig. 6A) revealed three distinct clusters: the cecum formed an isolated cluster, the ileum and colorectum clustered together, and the remaining five compartments (liver, bile, serum, duodenum, and jejunum) formed a third cluster, though with some spread. PERMANOVA pairwise comparisons (Fig. 6B) confirmed that the cecal BA profile differed significantly from all other compartments (*P*-adj < 0.05), whereas the ileum and colorectum showed no significant difference between each other (*P*-adj > 0.05). Within the third cluster, there were no significant differences in the BA profiles of the liver and bile, serum and bile, or the duodenum and jejunum (Fig. 6B; *P*-adj > 0.05). All other pairwise comparisons among these five compartments were statistically significant (*P*-adj < 0.05). These five compartments are all characterized by a high proportion of taurine-conjugated primary BAs, particularly TCDCA (Fig. S3). The spatial distribution of BA classifications is shown in Fig. 6C–G. At d 21, the proportion of unconjugated BAs differed significantly among compartments (Fig. 6C, *P* < 0.05). The ileum (83.92% ± 2.22%) and colorectum (77.12% ± 4.16%) had significantly higher unconjugated BA proportions than the cecum (50.35% ± 4.19%; *P* < 0.05), whereas all other compartments showed very low proportions (< 10%). Conversely, conjugated BAs dominated in the liver, bile, serum, duodenum, and jejunum (proportions > 90%), with significantly lower levels in the ileum, cecum, and colorectum (Fig. 6D, *P* < 0.05). Primary BAs accounted for the majority in all compartments, but their proportion was significantly lower in the cecum (70.82 ± 5.58%) than in all other compartments (> 96%; Fig. 6E, *P* < 0.05). Secondary BAs were significantly enriched in the cecum compared with all other compartments (Fig. 6F, *P* < 0.05), with LCA being the predominant secondary BA (Fig. S3). The ratio of 12‑OH to non‑12‑OH BAs also varied significantly across compartments (Fig. 6G, *P* < 0.05). The duodenum, jejunum, and liver shared the highest ratio, followed by the cecum and bile, whereas the ileum, serum, and colorectum had the lowest ratios.

**Fig. 7:**
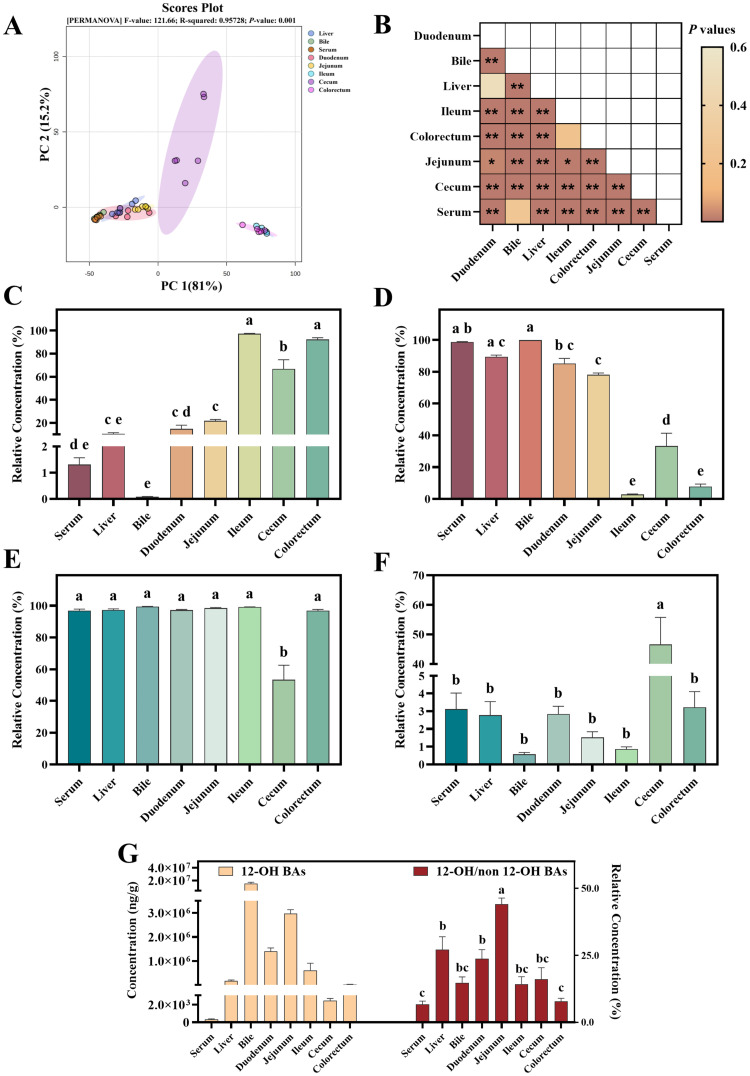
**The spatial heterogeneity of bile acids across the liver-gut axis samples in 42-day-old broilers**. (A) Principal component analysis (PCA) plot with 95% confidence ellipses depicting the overall compositional separation of bile acid profiles. (B) Heatmap of pairwise PERMANOVA *P*-values comparing bile acid composition between all sample types, * *P* < 0.05, ** *P* < 0.01. (C-F) Bar graphs showing the relative concentrations of key bile acid classifications in the samples: (C) unconjugated, (D) conjugated, (E) primary, and (F) secondary bile acids. (G) The concentrations of 12α-hydroxylated (12-OH BAs) and the relative proportions of 12-OH BAs and non-12α-hydroxylated bile acids (non-12OH BAs). For panels (C) through (G), different lowercase letters (a, b, c) above the bars indicate significant differences among sample types (*P* < 0.05, one‑way ANOVA followed by Tukey’s HSD test). Values sharing the same letter are not significantly different. Data are presented as mean ± SEM (*n* = 6).

At d 42, significant spatial heterogeneity of the BA pool was also observed (Fig. 7A; F = 121.66, *P* = 0.001). Similar to d 21, the PCA plot (Fig. 7A) revealed three distinct clusters: the cecum formed an isolated cluster; the ileum and colorectum clustered together; and the remaining five compartments (liver, bile, serum, duodenum, and jejunum) formed a third cluster, though with some spread. PERMANOVA pairwise comparisons (Fig. 7B) confirmed that the cecal BA profile differed significantly from all other compartments (*P*-adj < 0.05), whereas the ileum and colorectum showed no significant difference between each other (*P*-adj > 0.05). Unlike the clear separation observed between ileum and colon BA profiles in mammalian species, this persistent clustering further confirms the avian-specific feature. Within the third cluster, there were no significant differences in the BA profiles of the duodenum and liver, or between bile and serum (Fig. 7B; *P*-adj > 0.05). Notably, the jejunum emerged as a distinct compartment. Although it shared some characteristics with the proximal cluster, its BA profile differed significantly from those of all other compartments (Fig. 7A–B, *P*-adj < 0.05). The spatial distribution of BA classifications is shown in Fig. 7C–G. At d 42, the proportion of unconjugated BAs differed significantly among compartments (Fig. 7C, *P* < 0.05). The ileum (97.23% ± 0.40%) and colorectum (92.21% ± 1.56%) had significantly higher unconjugated BA proportions than the cecum (66.67% ± 8.07%; *P* < 0.05). Conversely, conjugated BAs dominated in the liver, bile, serum, duodenum, and jejunum (proportions > 78%), with particularly high proportions in serum (98.69% ± 0.26%) and bile (99.92% ± 0.01%; Fig. 7D, *P* < 0.05). Primary BAs accounted for over 96% of the total in all compartments except the cecum (Fig. 7E). Secondary BAs were significantly enriched in the cecum (46.61% ± 9.14%) compared with all other compartments (Fig. 7F, *P* < 0.05). The ratio of 12‑OH to non‑12‑OH BAs also varied significantly across compartments (Fig. 7G, *P* < 0.05). The jejunum exhibited the highest ratio (44.11% ± 2.27%), whereas all other compartments had ratios below 30%, with serum and colorectum below 8%.

## Discussion

The enterohepatic circulation of BAs is a dynamic and highly regulated process that is integral to lipid digestion, metabolic signalling and host-microbiota communication (Sutton et al., 2024). This study provides a comprehensive spatiotemporal mapping of the BAs pool across eight key compartments in broilers, from hatching to reaching market age. The liver is the primary organ responsible for BA synthesis and determines the systemic BA pool; its BA profile also serves as a key indicator of hepatic metabolic health (Wuethrich et al., 2021). The gallbladder acts as the main BAs reservoir, with its profile directly reflecting hepatic synthesis and biliary secretion (Cao et al., 2024). In the small intestine, BAs are essential for the digestion and absorption of lipids and act as signalling molecules in feedback regulation of hepatic synthesis (Huang et al., 2021). The duodenum is the initial site of nutrient digestion, while the jejunum is a key location for fat emulsification. Thus, their BA profiles reflect the functional state during digestion (Bai et al., 2025). The ileum is the main site of active BA reabsorption, and its profile reflects recycling efficiency and preliminary microbial transformation. In broilers, the paired cecum opens at the junction of the ileum and the colorectum (Clavijo and Flórez, 2018). Due to this anatomical structure, only a small amount of digesta enters the cecum, while the majority flows directly into the colorectum for excretion. The cecum functions as a primary fermentation chamber, hosting a dense microbial community that extensively modifies BAs via deconjugation, 7α‑dehydroxylation, and isomerisation; consequently, the cecal BA profile carries a distinct signature of specialised microbial metabolism. The colorectum BA profile reflects the final excretory characteristics of the BA pool, and the serum BA profile represents a dynamic equilibrium of intestinal absorption, hepatic uptake, and biliary secretion. Characterizing BA profiles across these functionally distinct compartments is necessary for understanding the developmental trajectory and tissue specificity of bile acid metabolism in broilers.

The concentration of TBA showed a consistent spatial hierarchy, with the highest concentration found in gallbladder bile, followed by the small intestinal lumen, the liver, colorectum and, finally, serum. This gradient emphasises the efficiency of hepatic concentration and gallbladder storage, as previously described in a published article (Green and Kellogg, 1987). The developmental trajectories of TBA in key organs revealed distinct patterns. A notable decrease in hepatic TBA concentration was observed during week 4 and week 5, before increasing again at week 6. In theory, increased dietary fat should require a greater amount of bile acids for emulsification and absorption (Poland and Flynn, 2021). However, rather than an immediate increase, hepatic TBA concentration declined during weeks 4 and 5. One possible explanation is that the sudden rise in secretory demand upon introduction of the high‑fat diet temporarily exceeds the liver’s synthetic capacity, leading to a net depletion of the hepatic BA pool. High‑fat diets are known to upregulate hepatic BA synthesis (Chiang, 2013), but this adaptive response may take time to become fully effective. By week 6, the compensatory increase in synthesis appears to have caught up with or exceeded the elevated secretory demand, resulting in a rebound of hepatic TBA concentration. This pattern suggests a transient lag between increased demand and full synthetic adaptation during the early phase of high‑fat feeding. The significant increase in duodenal TBA concentration by week 3 occurred without concurrent changes in hepatic or biliary TBA concentrations. This suggests an age‑related alteration in the dynamics of bile flow or intestinal transit, rather than a change in overall BA synthesis or storage. Direct evidence is lacking and further studies are needed to clarify the underlying mechanisms. Jack Green et al. (Green and Kellogg, 1987) discovered that broiler chickens had already accumulated a certain amount of BAs by the age of two days. Similar to the existing results (Lundell and Wikvall, 2008), our data also indicate that the biosynthesis of BAs may occur in the early stages of life.

The balance between 12-OH BAs and non-12-OH BAs is central to the functionality of the BA pool (Fan et al., 2025). The hepatic enzyme cytochrome P450 8B1 (CYP8B1) is the key rate-limiting enzyme in the synthesis of 12-OH BAs, and thus directly determines the ratio (Hori et al., 2020). In our study, the significant increase in 12-OH BA concentration in the duodenum at week 3, despite no change in the 12-OH to non-12-OH ratio, is consistent with the elevated overall BA output observed during this period. This suggests that the increase is proportional across BA classifications, rather than a shift in synthetic preference. Our data revealed that, although the ratio of 12-OH to non-12-OH BA remained stable over time, its spatial distribution was significant. The highest ratio was observed in the jejunum, the primary site for active nutrient absorption. This suggests an enhanced role for 12-OH BAs in lipid digestion. A higher proportion of 12-OH BAs is generally associated with greater lipid absorption efficiency (Li et al., 2021; Oteng et al., 2021). 12-OH BAs (TCA and glycocholic acid [GCA]) facilitate the differential solubilisation of fatty acids into mixed micelles, with greater efficiency for unsaturated fatty acids, particularly polyunsaturated fatty acids (Chan et al., 2025). A study of piglets showed that an increased proportion of 12-OH BAs in the jejunum potentially facilitates intestinal absorption (Mao et al., 2025). Wang et al. (2024) observed that non-12-OH BAs may play a crucial role in the lipid metabolism of the liver in broilers. Supplementing non-12-OH BAs might reduce fat deposition in the liver of broilers. Our study provides a comprehensive baseline of the spatial and temporal distribution of 12-OH BAs in broilers, which may serve as a foundation for future research on their nutritional regulation.

Our longitudinal bile acid data provide indirect evidence for the functional maturation of the gut microbiota in broilers. A dramatic and irreversible transformation of conjugated BAs into unconjugated BAs occurred in the ileum by week 3. This reaction is catalyzed by microbial BSH, which cleaves the conjugated BAs. BSH is considered the gateway enzyme for further microbial modification of bile acids, such as 7α‑dehydroxylation (Fu et al., 2025). Importantly, this transition took place while the broilers were still receiving the low‑fat starter diet, before the switch to the high‑fat grower diet. Thus, the increased deconjugation cannot be explained by the change of dietary fat level, although we cannot exclude the possibility that dietary fat modulates BSH activity later in life. The observation may suggest that age‑related maturation of the BSH‑active microbial community is a sufficient driver of this process. Conjugated bile acids, such as TCDCA, have a lower critical micellar concentration and greater emulsifying capacity than their unconjugated forms (Hofmann and Mysels, 1992). Thus, deconjugation may initially reduce the efficiency of fat emulsification. In the cecum, a different set of microbial enzymes, the 7α‑dehydroxylases, further transform primary bile acids such as CDCA into secondary bile acids, most notably LCA. Accordingly, secondary BAs (particularly LCA) progressively accumulate in the cecum from week 3 onward. This sequential change—first deconjugation in the ileum and then 7α‑dehydroxylation in the cecum—provides functional readouts of gut microbiota maturation. Collectively, our data suggest that week 3 may represent a critical window for the acquisition of key microbial BA‑metabolizing activities, which occurs independently of the dietary fat shift and is more likely driven by host development and microbial succession.

We selected 21 and 42 days as the comparative time points. Our analyses revealed a sophisticated functional zonation along the gut-liver axis. At both time points, there is a clear demarcation between a host-dominant anabolic and secretion zone, and a microbiota-dominant catabolic and transformation zone. The former comprises the liver, bile, serum, duodenum and jejunum, and is characterised by a conserved profile of taurine-conjugated primary BAs (TCDCA and TCA). The significant reduction in TCDCA concentration from bile to duodenal contents suggests dilution and potential microbial deconjugation activity immediately upon intestinal entry. The catabolic and transformation zone includes the ileum, cecum, and colorectum. The similarity between ileal and colorectum BA profiles, coupled with the low proportion of secondary BAs in colorectum compared to the cecum, is a direct consequence of avian digestive anatomy. A significant proportion of digesta bypasses the cecum, meaning that excreted colorectum primarily reflects ileal content that has undergone limited further microbial modification (Clavijo and Flórez, 2018). The similarity between the BA profiles of the ileum and colorectum in broilers reflects avian digestive anatomy. In contrast, the ileum and colon exhibit distinct BA profiles in mammals due to extensive microbial fermentation. In broilers, the cecum is a unique fermentation zone, distinct from all other sites. The cecum's BA profile is rich in secondary BAs, such as LCA. The cecum exhibited a significantly higher proportion of glycine-conjugated BAs compared to other compartments, suggesting that BSH has aminoacyl transferase activity in the intestine to generate microbial conjugated BAs (Guzior et al., 2024).

The BA pool in broilers is shaped by a dynamic interplay between the host and gut microbiota. BAs have antimicrobial effects that help shape the structure of microbial communities (Strathearn et al., 2020). In return, the microbiota remodels the BA pool extensively through deconjugation, epimerisation and dehydroxylation, creating a spatially defined pool (Sagar et al., 2015; Jia and Dong, 2024). The balance between conjugated and unconjugated BAs is crucial, as it influences BA signalling through receptors like FXR and their antimicrobial potency (Liu et al., 2015). Research in murine models has shown that the equilibrium between BA conjugation and hydrolysis can affect the outcome of gut inflammation (Fu et al., 2025). While our study reveals distinct spatial variations in deconjugation efficiency between the jejunum and ileum in broilers, the specific impact on gut health remains to be elucidated. The significantly decreased ratio of 12-OH to non-12-OH BAs in the ileum, compared with the jejunum, reflects the specificity of BA reabsorption efficiency, which could be key to modulating their specific physiological effects (Cai et al., 2024).

The developmental and spatial patterns described in this study have direct physiological implications. The data from this study reflect the gradual maturation of BSH and 7α‑dehydroxylase activities in broilers. BSH activity becomes prominent in the ileum by week 3. This activity liberates unconjugated BAs from their conjugated forms. Unconjugated BAs are less efficient at emulsifying dietary lipids (Łozińska et al., 2024), but their increased abundance may signal to the host via the gut-liver axis to modulate BA synthesis and enterohepatic circulation (Hartmann et al., 2018). Furthermore, deconjugation is a prerequisite for subsequent 7α-dehydroxylation (Fu et al., 2025). This intensifies in the cecum from week 3 onwards, converting primary bile acids (e.g. CDCA) into secondary bile acids (e.g. LCA). This transformation reduces the solubility and absorptive capacity of the BA pool (Samartsev et al., 2023). From a nutritional perspective, the timing of these microbial events coincides with the broiler’s rapid growth phase and transition to a higher-fat diet (day 21). While the enhanced microbial deconjugation and dehydroxylation may initially reduce fat digestion efficiency, the adaptive increase in BA synthesis and pool size (reflected by the rebound in hepatic TBA concentration at week 6) likely compensates for this to maintain overall lipid absorption. Meanwhile, the progressive increase in the proportion of LCA in the cecum and colorectum may create a less permissive luminal environment for harmful bacteria, thereby supporting natural resistance against enteric pathogens (Ma et al., 2023). Dietary strategies employing prebiotics, probiotics or bioactive compounds could be designed to modulate BA composition, potentially optimising nutrient absorption, gut barrier function or systemic metabolism (Li et al., 2026).

A primary limitation of this study is the lack of parallel microbiome sequencing data, which prevents the direct correlation of BA transformations (e.g., deconjugation, 7α‑dehydroxylation) with specific bacteria. Additionally, this study was conducted only with male broilers. Therefore, potential sexual dimorphism in BA metabolism was not addressed. Future research should adopt a multi-omics approach to establish causative links and include both sexes to evaluate sex‑specific differences in BA dynamics.

## Conclusion

In conclusion, this study reveals the spatiotemporal development of the BA pool in broilers. First, TBA concentrations exhibit a consistent spatial gradient along the gut‑liver axis, with the highest concentrations in bile and the lowest in serum. Second, based on the age‑dependent changes in BA profiles, week 3 post‑hatching appears to be a critical window for the functional maturation of microbial bile acid metabolism, marked by efficient deconjugation in the ileum and 7α‑dehydroxylation in the cecum. Third, the striking similarity between ileal and colorectal BA profiles represents an avian‑specific trait, contrasting with the distinct profiles observed in mammals. These findings provide a fundamental framework for understanding broiler digestive physiology and offer insights for developing nutritional strategies to optimise gut health and production efficiency by targeting BA metabolism.

## CRediT authorship contribution statement

**Sunlin Luo:** Writing – original draft, Visualization, Project administration, Data curation. **Ruiqi Tan:** Investigation, Data curation. **Qiaomin Duan:** Data curation. **Wenjun He:** Data curation. **Ying Liu:** Validation, Project administration, Methodology. **Yiqiang Chen:** Writing – review & editing, Funding acquisition, Conceptualization.

## Disclosures

All authors disclosed no relevant relationships.

## Appendix Supplementary materials

Image, application 1

## Footnotes

Supplementary material associated with this article can be found, in the online version, at doi:10.1016/j.psj.2026.107353.
