# Supplementary material for: Spatiotemporal dynamics of bile acid profiles in broilers from hatch to market age
Source: Poult Sci. 2026 Jun 27;105(10):107353. doi: 10.1016/j.psj.2026.107353 (PMC13356759; doi:10.1016/j.psj.2026.107353)
Supplement: Supplementary file 1 [file mmc1.docx]

Supplemental material

**Spatiotemporal dynamics of bile acid profiles in broilers from hatch to market age**

Sunlin Luo, Ruiqi Tan, Qiaomin Duan, Wenjun He, Ying Liu, Yiqiang Chen ^*^

*State Key Laboratory of Animal Nutrition and Feeding, College of Animal Science and Technology, China Agricultural University, Beijing 100193, China.*

**^*^ Corresponding author:**

Yiqiang Chen, Professor; E-mail: [yqchen@cau.edu.cn](mailto:yqchen@cau.edu.cn)

**Table S1** Detection parameters and classification of 28 bile acids

| Bile acids | Fragmentor /V | Collision energy /eV | Qualitative ion pair/(m/z) | Retention time/min | Internal standard | Limit of quantitation/(ng/mL) | Classification |
| --- | --- | --- | --- | --- | --- | --- | --- |
| Lithocholic acid (LCA) | 250 | 10 | 375.3/375.3 | 24.775 | LCA-d4 | 2.0 | unconjugated, secondary, non-12α-hydroxylated |
| Chenodeoxycholic acid (CDCA) | 250 | 10 | 391.3/391.3 | 23.712 | DCA-d4 | 2.3 | unconjugated, primary, non-12α-hydroxylated |
| Deoxycholic acid (DCA) | 250 | 10 | 391.3/391.3 | 23.826 | DCA-d4 | 0.7 | unconjugated, secondary, 12α-hydroxylated |
| Ursodeoxycholic acid (UDCA) | 250 | 10 | 391.3/391.3 | 22.614 | DCA-d4 | 0.7 | unconjugated, secondary, non-12α-hydroxylated |
| Hydroxycholic acid (HDCA) | 250 | 10 | 391.3/391.3 | 22.732 | DCA-d4 | 0.7 | unconjugated, secondary, non-12α-hydroxylated |
| Cholic acid (CA) | 250 | 10 | 407.3/407.3 | 22.557 | CA-d4 | 2.0 | unconjugated, primary, 12α-hydroxylated |
| α-Muricholic acid (α-MCA) | 250 | 10 | 407.3/407.3 | 19.966 | CA-d4 | 3.3 | unconjugated, primary, non-12α-hydroxylated |
| β-Muricholic acid (β-MCA) | 250 | 10 | 407.3/407.3 | 20.237 | CA-d4 | 2.0 | unconjugated, primary, non-12α-hydroxylated |
| Hyocholic acid (HCA) | 250 | 10 | 407.3/407.3 | 21.926 | CA-d4 | 1.0 | unconjugated, primary, non-12α-hydroxylated |
| ω-Muricholic acid (ω-MCA) | 250 | 10 | 407.3/407.3 | 19.571 | CA-d4 | 2.3 | unconjugated, secondary, non-12α-hydroxylated |
| Glyclithocholic acid (GLCA) | 200 | 40 | 432.3/74.1 | 23.952 | GLCA-d5 | 0.7 | conjugated, secondary, non-12α-hydroxylated |
| Glycochenodeoxycholic Acid (GCDCA) | 200 | 40 | 448.3/74.1 | 22.792 | GDCA-d4 | 0.7 | conjugated, primary, non-12α-hydroxylated |
| Glycodeoxycholic acid (GDCA) | 200 | 40 | 448.3/74.1 | 23.003 | GDCA-d4 | 1.0 | conjugated, secondary, 12α-hydroxylated |
| Glycoursodeoxycholic acid (GUDCA) | 200 | 40 | 448.3/74.1 | 19.745 | GUDCA-d4 | 3.3 | conjugated, secondary, non-12α-hydroxylated |
| Glycohydroxycholic acid (GHDCA) | 200 | 40 | 448.3/74.1 | 20.063 | GUDCA-d4 | 3.3 | conjugated, secondary, non-12α-hydroxylated |
| Glycocholic acid (GCA) | 200 | 40 | 464.3/74.1 | 20.19 | GCA-d5 | 0.7 | conjugated, primary, 12α-hydroxylated |
| Glyco-β-muricholic acid (Gβ-MCA) | 200 | 40 | 464.3/74.1 | 16.835 | GCA-d5 | 3.3 | conjugated, primary, non-12α-hydroxylated |
| Glycohyocholic acid (GHCA) | 200 | 40 | 464.3/74.1 | 18.585 | GCA-d5 | 0.7 | conjugated, primary, non-12α-hydroxylated |
| Taurolithocholic acid (TLCA) | 260 | 80 | 482.3/80.1 | 23.643 | TLCA-d5 | 0.7 | conjugated, secondary, non-12α-hydroxylated |
| Taurochenodeoxycholic acid (TCDCA) | 260 | 80 | 498.3/80.1 | 21.806 | TCDCA-d5 | 3.3 | conjugated, primary, non-12α-hydroxylated |
| Taurodeoxycholic acid (TDCA) | 260 | 80 | 498.3/80.1 | 22.319 | TCDCA-d5 | 3.3 | conjugated, secondary, 12α-hydroxylated |
| Tauroursodeoxycholic acid (TUDCA) | 260 | 80 | 498.3/80.1 | 17.737 | TCDCA-d5 | 0.7 | conjugated, secondary, non-12α-hydroxylated |
| Taurohydroxycholic acid (THDCA) | 260 | 80 | 498.3/80.1 | 17.907 | TCDCA-d5 | 3.3 | conjugated, secondary, non-12α-hydroxylated |
| Taurocholic acid (TCA) | 260 | 80 | 514.3/80.1 | 18.424 | TCA-d5 | 1.0 | conjugated, primary, 12α-hydroxylated |
| Tauro-α-Muricholic acid (Tα-MCA) | 260 | 80 | 514.3/80.1 | 14.7 | TCA-d5 | 3.3 | conjugated, primary, non-12α-hydroxylated |
| Tauro-β-Muricholic acid (Tβ-MCA) | 260 | 80 | 514.3/80.1 | 14.818 | TCA-d5 | 3.3 | conjugated, primary, non-12α-hydroxylated |
| Taurohyocholic acid (THCA) | 260 | 80 | 514.3/80.1 | 16.582 | TCA-d5 | 3.3 | conjugated, primary, non-12α-hydroxylated |
| Tauro-ω-Muricholic acid (Tω-MCA) | 260 | 80 | 514.3/80.1 | 14.428 | TCA-d5 | 3.3 | conjugated, secondary, non-12α-hydroxylated |


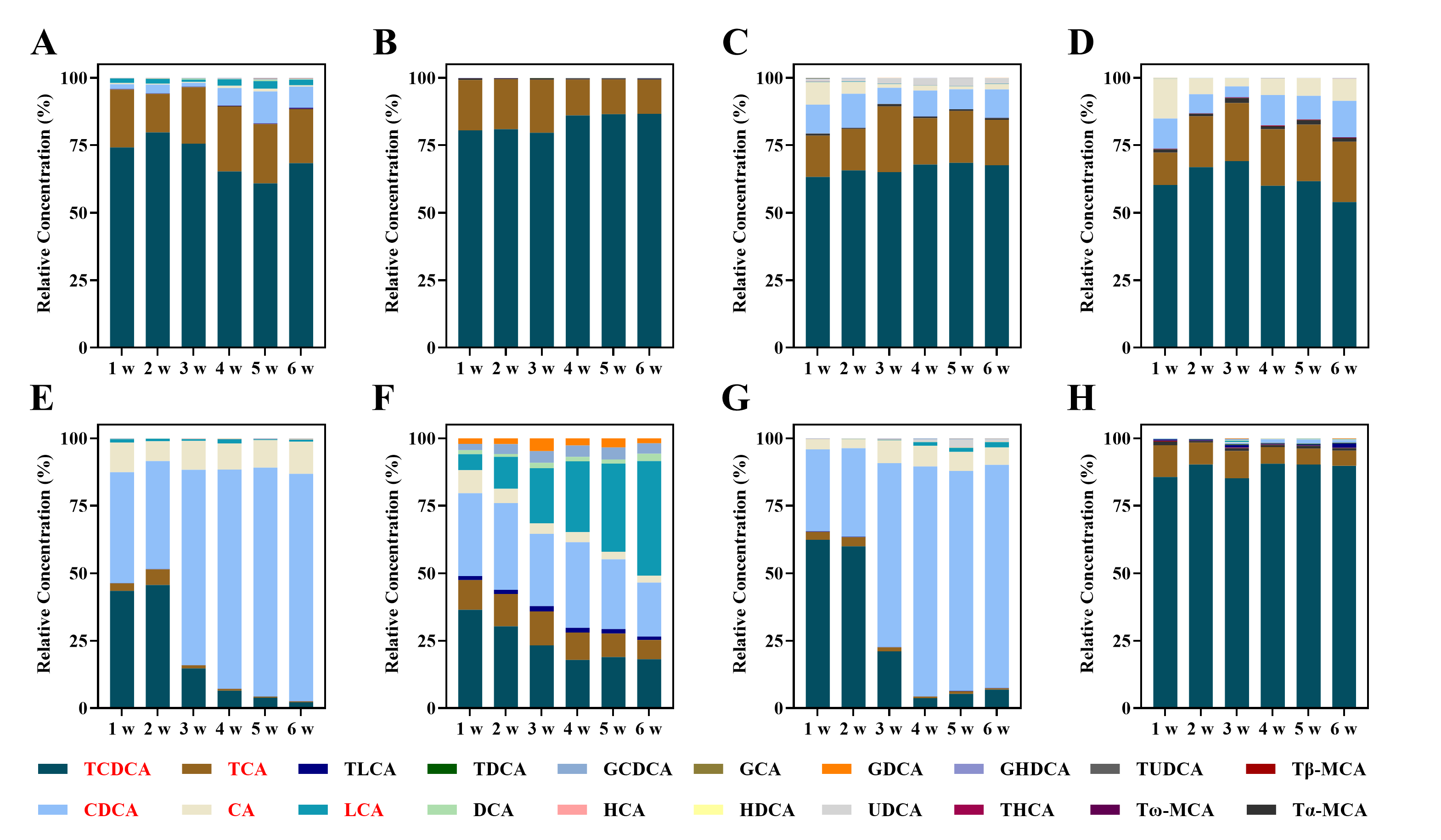


Fig. S1. Dynamic changes of bile acid profiles in broiler tissues and biofluids across weeks of age. (A) Liver, (B) Bile, (C) Duodenum, (D) Jejunum, (E) Ileum, (F) Cecum, (G) Colorectum, and (H) Serum. *n* = 6. Chenodeoxycholic acid: CDCA, Taurochenodeoxycholic acid: TCDCA, Taurocholic acid: TCA, Cholic acid: CA, Taurolithocholic acid: TLCA, Lithocholic acid: LCA, Taurodeoxycholic acid: TDCA, Deoxycholic acid: DCA, Glycochenodeoxycholic acid: GCDCA, Hyocholic acid: HCA, Glycocholic acid: GCA, Hyodeoxycholic acid: HDCA, Glycodeoxycholic acid: GDCA, Ursodeoxycholic acid: UDCA, Taurohyocholic acid: THCA, Glycohyodeoxycholic acid: GHDCA, Tauroursodeoxycholic acid: TUDCA, Tauro-ω-muricholic acid: TωMCA, Tauro-β-muricholic acid: TβMCA, Tauro-α-muricholic acid: TαMCA. Note: bile acid species that account for >5% of the total proportion in at least one tissue (TCDCA, TCA, CDCA, CA, and LCA) are shown in red font, whereas all other bile acid species are shown in black font.


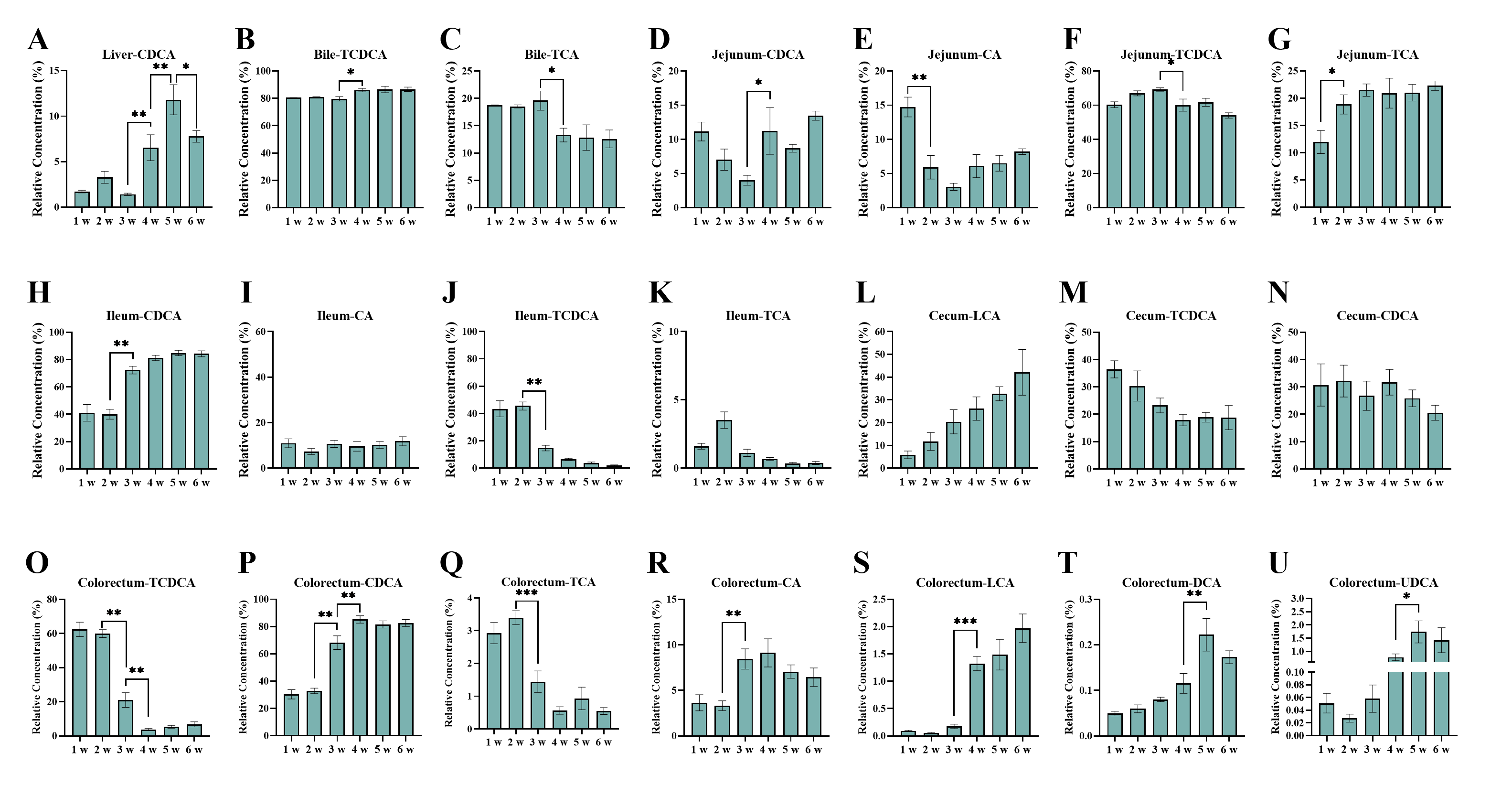


Fig. S2. The weekly changing trends of key bile acids in broiler. (A): Liver; (B-C): Bile; (D-G): Jejunum; (H-K): Ileum; (L-N): Cecum; (O-U): colorectum. Chenodeoxycholic acid : CDCA, Taurochenodeoxycholic acid : TCDCA, Taurocholic acid : TCA, Cholic acid : CA, Lithocholic acid : LCA, Deoxycholic acid : DCA, Ursodeoxycholic acid: UDCA . For each tissue, one‑way ANOVA with Bonferroni‑adjusted pairwise comparisons was performed between consecutive weeks. Data are presented as mean ± SEM (*n* = 6).* *P* < 0.05, ** *P* < 0.01, *** *P* < 0.001.


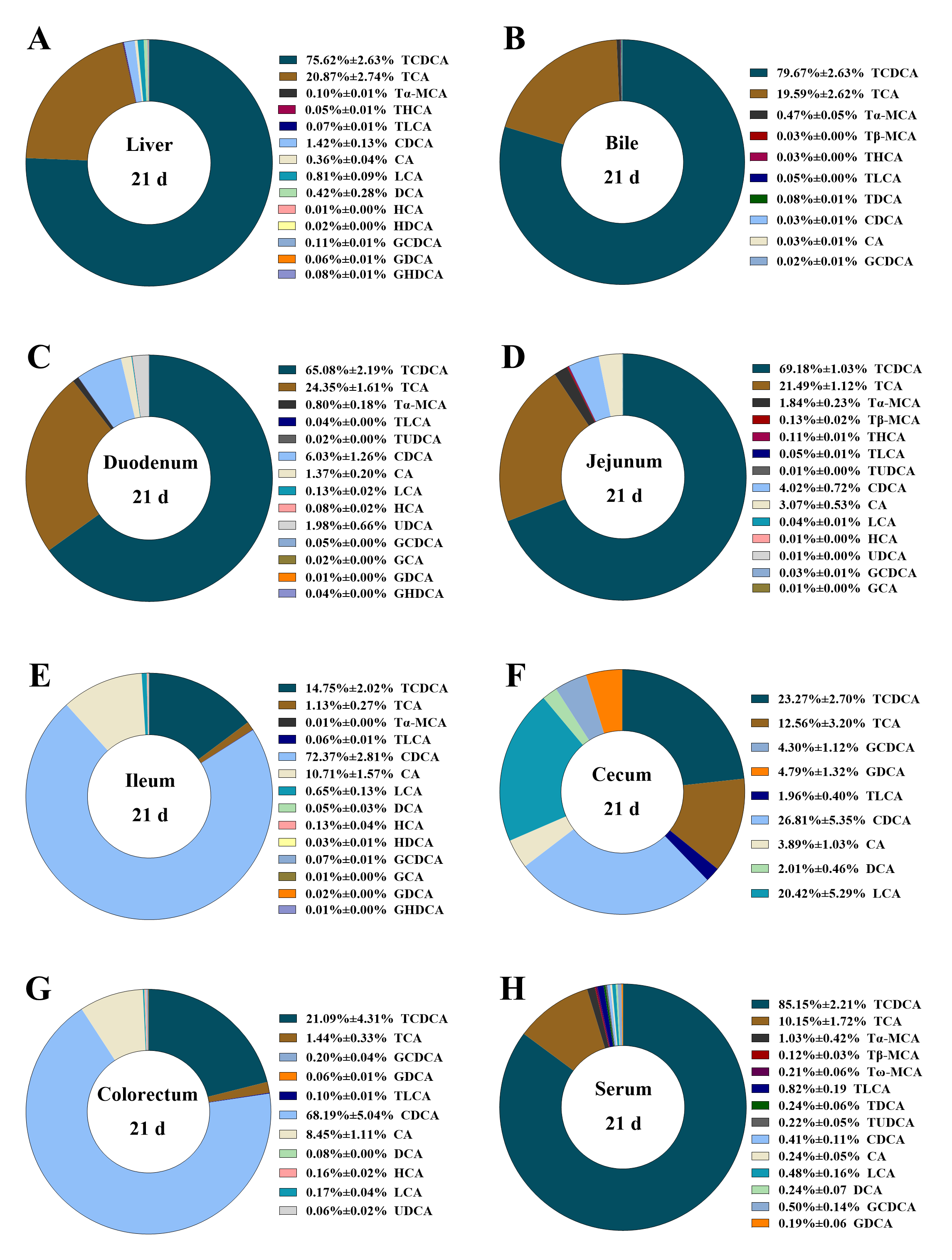


Fig. S3. The bile acid profiles of 21-day-old broilers. (A) Liver, (B) Bile, (C) Duodenum, (D) Jejunum, (E) Ileum, (F) Cecum, (G) Colorectum, and (H) Serum.


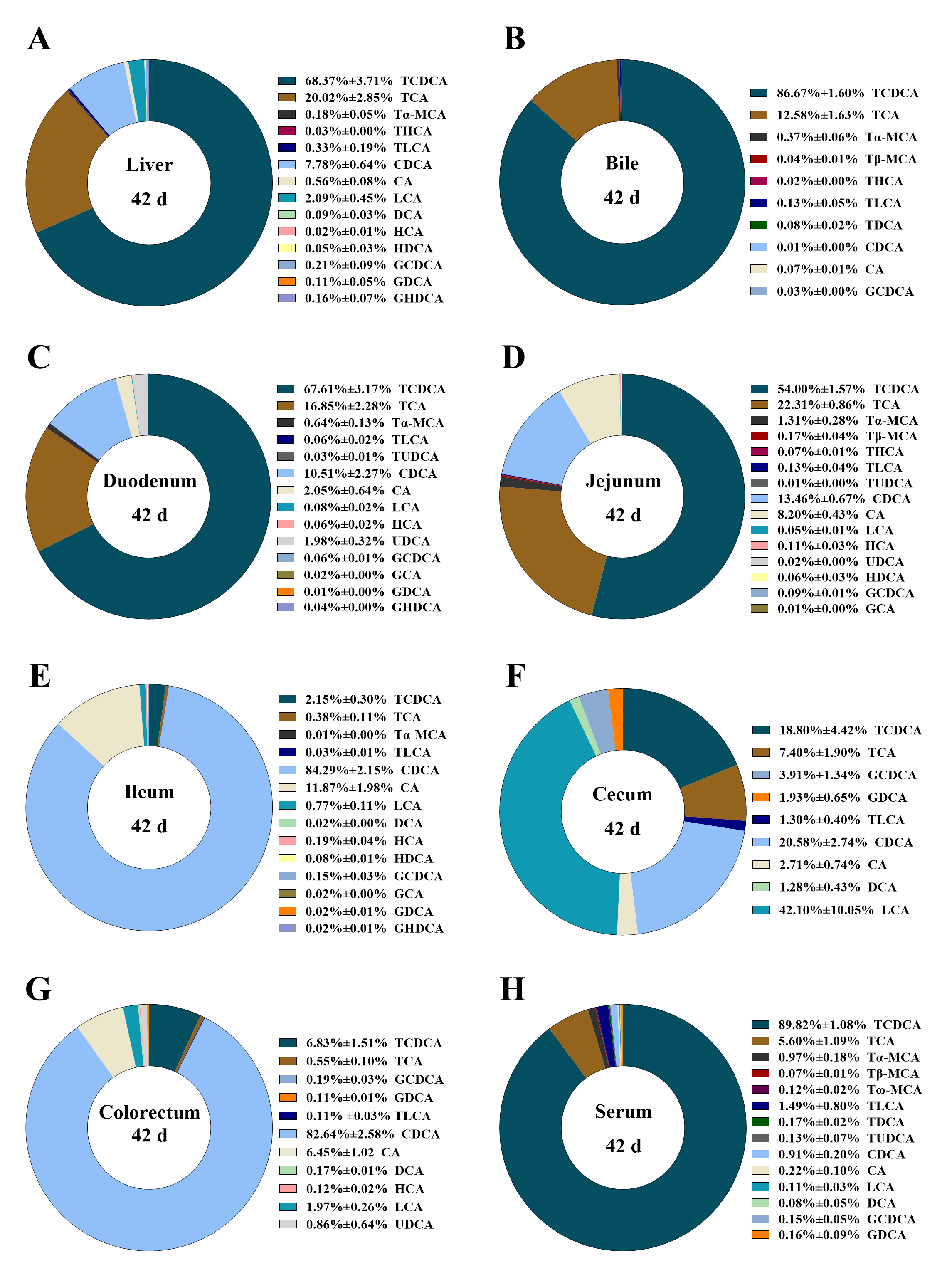


Fig. S4. The bile acid profiles of 42-day-old broilers. (A) Liver, (B) Bile, (C) Duodenum, (D) Jejunum, (E) Ileum, (F) Cecum, (G) Colorectum, and (H) Serum.
